# Aerobic capacities and swimming performance of polar cod (*Boreogadus saida*) under ocean acidification and warming conditions

**DOI:** 10.1242/jeb.184473

**Published:** 2018-10-31

**Authors:** Kristina Lore Kunz, Guy Claireaux, Hans-Otto Pörtner, Rainer Knust, Felix Christopher Mark

**Affiliations:** 1 Alfred Wegener Institute Helmholtz Centre for Polar and Marine Research, Bentho-Pelagic Processes, Am Alten Hafen 26, 27568 Bremerhaven, Germany; 2 Alfred Wegener Institute Helmholtz Centre for Polar and Marine Research, Integrative Ecophysiology, Am Handelshafen 12, 27570 Bremerhaven, Germany; 3 University of Bremen, Fachbereich 2, NW 2/Leobener Strasse, 28359 Bremen, Germany; 4 Université de Bretagne Occidentale, LEMAR (UMR 6539), Unité PFOM, Laboratoire ARN, Centre Ifremer de Brest, 29280 Plouzané, France

**Keywords:** Climate change, Gadids, Arctic cod, Hypercapnia, RCP8.5, Aerobic scope

## Abstract

Polar cod (*Boreogadus saida*) is an important prey species in the Arctic ecosystem, yet its habitat is changing rapidly: climate change, through rising seawater temperatures and CO_2_ concentrations, is projected to be most pronounced in Arctic waters. This study aimed to investigate the influence of ocean acidification and warming on maximum performance parameters of *B. saida* as indicators for the species' acclimation capacities under environmental conditions projected for the end of this century. After 4 months at four acclimation temperatures (0, 3, 6, 8°C) each combined with two *P*_CO_2__ levels (390 and 1170 µatm), aerobic capacities and swimming performance of *B. saida* were recorded following a *U*_crit_ protocol. At both CO_2_ levels, standard metabolic rate (SMR) was elevated at the highest acclimation temperature indicating thermal limitations. Maximum metabolic rate (MMR) increased continuously with temperature, suggesting an optimum temperature for aerobic scope for exercise (AS_ex_) at 6°C. Aerobic swimming performance (*U*_gait_) increased with acclimation temperature irrespective of CO_2_ levels, while critical swimming speed (*U*_crit_) did not reveal any clear trend with temperature. Hypercapnia evoked an increase in MMR (and thereby AS_ex_). However, swimming performance (both *U*_gait_ and *U*_crit_) was impaired under elevated near-future *P*_CO_2__ conditions, indicating reduced efficiencies of oxygen turnover. The contribution of anaerobic metabolism to swimming performance was very low overall, and further reduced under hypercapnia. Our results revealed high sensitivities of maximum performance parameters (MMR, *U*_gait_, *U*_crit_) of *B. saida* to ocean acidification. Impaired swimming capacity under ocean acidification may reflect reduced future competitive strength of *B. saida*.

## Abstract (precis)

**Summary:** Polar cod is a key species in the Arctic ecosystem; its swimming capacity decreases under future water conditions, likely reducing its survival.

## INTRODUCTION

The oceans are currently experiencing a warming trend in parallel with increasing *P*_CO_2__ levels (Caldeira and Wickett, 2003; IPCC, 2014). These changes are expected to be fastest in Arctic waters due to the high solubility of CO_2_ in cold waters (Fransson et al., 2009), and an increase in the temperature of Atlantic water masses flowing into the Arctic ocean (Polyakov et al., 2010). This accelerates the decline in sea-ice cover and the freshening of surface waters (McPhee et al., 1998), which, in turn, exacerbates ocean acidification due to decreasing buffer capacities (Steinacher et al., 2009). According to the Representative Concentration Pathway representing business-as-usual CO_2_ emissions (RCP 8.5), the Arctic is projected to experience a rise in surface temperatures of 4–11°C by the year 2100 compared with the period 1986–2005 (IPCC, 2014). Within the same timeframe, *P*_CO_2__ levels in the Arctic ocean are projected to rise from 400 µatm to up to 1370 µatm (IPCC, 2014). Warming and potentially other climate change stressors such as ocean acidification appear to be already causing large-scale geographic shifts of marine species (Poloczanska et al., 2014) such as the ongoing borealization of the Arctic (Fossheim et al., 2015), entailing significant effects on the Arctic food chain.

Temperature is considered to be the most important abiotic factor shaping the geographical distribution of aquatic species (Magnuson et al., 1979; Perry et al., 2005; Fossheim et al., 2015) because of its effects on biochemical and physiological processes (Reynolds and Casterlin, 1979; Pörtner and Farrell, 2008). Accordingly, ectotherms tolerate a range of species-specific habitat temperatures that support the functionality of their molecular, cellular and systemic processes (Pörtner and Farrell, 2008). The species' thermal performance window can be understood from the ability of aerobic metabolic capacities to cover higher-than-baseline maintenance costs (Pörtner, 2010), as exemplified by aerobic scope (AS) [maximum metabolic rate (MMR)−standard metabolic rate (SMR)]. Within the thermal window, aerobic scope increases towards a species-specific optimum temperature and decreases rapidly at thermal conditions exceeding the optimum (Pörtner and Farrell, 2008; Farrell, 2016; Pörtner et al., 2017). Thermal performance windows are delimited by upper and lower critical temperatures at which aerobic scope reaches zero, solely supporting a time-limited passive and anaerobic existence (Pörtner and Farrell, 2008). In contrast, maximized aerobic scope at the species-specific optimum temperature implies optimum conditions for the performance of a given activity (Fry, 1947). Assuming that different aerobic activities do not necessarily have identical optimum temperatures, a broad thermal window with maximum aerobic scope covering a wide thermal range implies reduced competition between aerobic activities (Farrell, 2016). Most polar species, however, are adapted and specialized to the low temperatures and low thermal fluctuations of their natural habitat by evolving mechanisms to maintain overall performance along with reduced tolerance to changing abiotic conditions as a trade-off (Pörtner et al., 2000, 2005). Even relatively small increments in temperature can therefore have a tremendous impact on their metabolic demand (Claireaux et al., 2000; Pörtner, 2010) entailing a rise in energy turnover with detrimental consequences for fitness and performance traits e.g. growth, reproduction and swimming capacity (Butler et al., 1992). Despite decreasing performance capacities, the thermal range between peak aerobic scope and upper critical temperature is considered to be a buffer against a future increase in water temperature (Farrell, 2016), for the case where northward distribution shifts triggered by the motivation to preserve organismic performance cannot fully compensate for ocean warming.

List of abbreviations and symbols

AS_ex_: aerobic scope of exercise
BC_max_: maximum burst count
BC_tot_: total number of bursts
BL: body length
BW: body weight
*E*_max_: efficiency of maximum swimming performance
*Ṁ*_O_2__: rate of oxygen consumption
MMR: maximum metabolic rate
OAW: ocean acidification and warming
*P*_CO_2__: partial pressure of carbon dioxide
pH_tot_: total pH
SMR: standard metabolic rate
*T*_C,max_: critical thermal limit
*T*_pej_: pejus temperature
TSB: time between *U*_gait_ and *U*_crit_ (‘time spent bursting’)
TSB_anaerob_: estimated proportion of anaerobic metabolism between *U*_gait_ and *U*_crit_
*U*: swimming speed
*U*_crit_: maximum swimming speed
*U*_gait_: transition speed from purely aerobic to partly anaerobic swimming (∼maximum aerobic swimming speed)
*U*_max_: highest speed maintained for full time interval
*v*: velocity increment

Polar cod (*Boreogadus saida*, Lepechin 1774), is the most abundant Arctic gadid (Mueter et al., 2016 and references therein) and it is regarded as a key species in Arctic ecosystems (Bain and Sekerak, 1978; Welch et al., 1993; Hop and Gjøsæter, 2013) because of its role as a link between lower and higher trophic levels (Lowry and Frost, 1981; Bradstreet, 1982; Welch et al., 1993). Furthermore, it is the most energy-rich prey organism in the Arctic food chain (Harter et al., 2013). In recent years, the abundance of *B. saida* has been found to decrease in its southern distribution area in the Barents Sea as a result of rising water temperatures (Eriksen et al., 2015). Throughout its life stages, *B. saida* prefers different thermal habitats. Spawning takes place in shallow waters above 0°C with a peak period in January and February (Ajiad et al., 2011). Pelagic 0-group *B. saida* prefer 2.0–5.5°C (Eriksen et al., 2015), while juveniles and non-spawning adults are either ice-associated (Lønne and Gulliksen, 1989) or found in deep water layers below 0°C (Falk-Petersen et al., 1986). A progressive distribution retreat of *B. saida*, evoked directly or indirectly by climate change, might have profound, cascading ecological consequences. In order to gauge ecosystem impacts caused by rapidly changing abiotic conditions, the assessment of sensitivities and acclimation capacities of key species such as *B. saida* to future climate scenarios is highly important.

The whole-animal SMR is an important parameter for the assessment of long-term survival because it integrates essential cellular and molecular energetic costs in the inactive organism at the respective environmental conditions (Chabot et al., 2016). Therefore, the SMR of *B. saida* has been identified over a range of acclimation temperatures in a number of studies (Holeton, 1974; Steffensen et al., 1994; Hop and Graham, 1995; Kunz et al., 2016a). Maximum respiratory performance of *B. saida* has been studied by Drost et al. (2016); however, aerobic swimming capacity as a fitness parameter has never been quantified in *B. saida*.

Furthermore, studies investigating performance capacities of *B. saida* exposed to combined climate drivers such as ocean acidification and warming (OAW) are still scarce. Recent studies investigated growth performance, feed consumption and SMR (Kunz et al., 2016a), laterality and spontaneous activity (Schmidt et al., 2017) and heart mitochondria performance (Leo et al., 2017) in juveniles as well as survival rates, SMR and morphology at hatch in early life stages (Flemming Dahlke, Daniela Storch and H.-O.P., unpublished data) under combined OAW conditions. We hypothesize that traits involving maximum performance will also be affected by ocean acidification, possibly even more so than routine functions, as the former may reflect limits to acclimatization. Therefore, the aim of the present study is to investigate the impact of long-term exposure to projected OAW scenarios on AS_ex_ and swimming performance of *B. saida* in light of its whole-animal acclimation capacities to future Arctic water conditions.

## MATERIALS AND METHODS

All procedures reported in the present study were in accordance with the ethical standards of the federal state of Bremen, Germany, and were approved under the reference number 522-27-22/02-00 (113).

### Fish

*B. saida* originated from Isfjorden and Kongsfjorden on the west coast of Spitsbergen. They were caught by RV Helmer Hanssen using bottom trawls at a depth of 120 m in January 2013. A fish-lift connected to the trawl (Holst and McDonald, 2000) protected the fish from injuries during trawling. The animals were then kept in the aquaria of Havbruksstasjonen i Tromsø AS (HiT) until April 2013, when they were transported to the laboratories of the Alfred Wegener Institute (AWI) in Bremerhaven.

### Experimental design

*B. saida* specimens were acclimated to different combinations of present and projected future ocean water conditions (temperature: 0, 3, 6, 8°C; *P*_CO_2__: 390 and 1170 µatm) for approximately 4 months. Each temperature/*P*_CO_2__ treatment comprised 12 individuals placed in 24 litre aquaria. The *P*_CO_2__ conditions for each treatment were generated in a common header tank (∼200 litres) which then provided identical conditions in each of the individual tanks. The setting of *P*_CO_2__ levels was accomplished by equilibration with mixtures of air and CO_2_ provided by an automated mass flow controller system (4 and 6 channel MFC system, HTK, Hamburg, Germany).

The distribution of individuals between treatments was done randomly. The acclimation to experimental temperatures took place gradually (max. temperature change: 1°C in 24 h), followed by establishing experimental *P*_CO_2__ conditions within 1 day, as soon as the desired experimental temperatures were reached. Light conditions were maintained at 12 h light:12 h dark throughout the experiment. Each fish was fed *ad libitum* every fourth day with formulated high-protein feed pellets (Amber Neptun, 5 mm, Skretting AS, Norway). For details on whole-animal parameters throughout the incubation period, see Kunz et al. (2016b).

### Water chemistry

Temperature, salinity and pH (cross-calibrated to total pH scale) were monitored once to twice a week in triplicate for every treatment in order to verify the stability of *P*_CO_2__ conditions, as described in Kunz et al. (2016a). The seawater carbonate chemistry was calculated in the program CO2SYS (Lewis and Wallace, 1998) based on the total dissolved inorganic carbon and the pH_tot_ values as listed in table 2 in Kunz et al. (2016a). The full water chemistry raw data of the incubation can be found in Schmidt et al. (2016).

### Swimming performance measurements

Two swim tunnels (30 litres; dimension working section: 46.5×13.5×14 cm, Loligo Systems ApS, Denmark) were used simultaneously to determine the swimming performance of *B. saida* (*n*=4–6 per treatment), enabling the measurement of up to 6 individuals per day in temperature-controlled rooms. The swim tunnels were supplied with pre-conditioned water from the header tank of the respective incubation treatment. For the time span of the experiment, the water conditions in the tunnels were maintained by permanent aeration with a gas mixture containing the respective CO_2_ levels. Aeration was maintained in the reservoir tank surrounding the swimming chamber. The swim chamber was kept in open mode to avoid decreasing O_2_ concentrations as well as temperature and *P*_CO_2__ fluctuations in the chamber. Permanent seawater exchange between the outer reservoir tank and the swim chamber was established by an aquarium pump (9.2 litres min^−1^). The desired velocity was translated from a control unit to a propeller in the swim chamber. A uniform velocity profile and a laminar flow were promoted by honeycomb-shaped plastic inserts. A flow sensor (Vane wheel flow sensor FA, Höntzsch Instruments, Waiblingen, Germany) placed in the centre of the working section of the swim tunnel was used to calibrate the water velocity to voltage output from the control unit.

The fish were transferred to the swim tunnel on the third day after feeding. The experiment was started after an average period of 3.5 h of animal adjustment to the system at a basic velocity of 1.4–2.2 BL s^−1^. The swim tunnel was covered with an opaque plastic curtain in order to minimize disturbance due to movements in the room. Following the initial period of adjustment, the velocity was slowly, but continuously increased to the mean start velocity per treatment of 2.4–2.8 BL s^−1^ (with respect to different size classes at different temperatures), depending on swim tunnel and fish size, with larger fish exposed to higher starting velocities to obtain similar relative velocities (BL s^−1^). According to the developed swim protocol, each velocity step was maintained for 11 min. At each velocity step, burst-and-coast (also known as kick-and-glide) swimming events were counted for 30 s after 5 and 10 min. This count aimed at determining the water velocity at gait transition (*U*_gait_) from steady to unsteady swimming mode. Following the second counting, water velocity was slowly increased by 1.9±0.3 cm s^−1^. The experiment ended when the fish were exhausted, defined by their physical contact to the grid for at least 30 s. The critical swimming speed (*U*_crit_) was adjusted according to the actual time spent at the maximal velocity as suggested by Brett (1964):
(1)
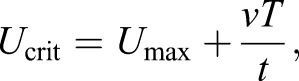
where *U*_max_ is highest velocity maintained for full time interval, *ν* is velocity increment, *T* is time spent at the velocity leading to fatigue and *t* is time interval. As soon as the fish gave up swimming, the velocity was rapidly decreased to the basic weaning velocity and fish were immediately transferred into respiration chambers. The short period of air exposure was used to weigh the fish to the nearest 0.1 g.

### Respiration measurements

Individual rates of oxygen consumption (*Ṁ*_O_2__ in µmol min^−1^ g^−1^) were measured at long-term acclimation temperature and *P*_CO_2__ by automated intermittent flow-through respirometry in a separate experimental set-up, comprising two sets of 6 perspex respiration chambers (1.8 and 2.2 litres). Respiration chambers were submerged as sets of two in common tanks (∼50 litres) with water conditions identical to the respective temperature/*P*_CO_2__ treatments. Partial water exchanges with pre-conditioned sea water were performed after ∼24 h. A non-transparent plastic wall between the respiration chambers prevented visual contact of the two individuals sharing a common water basin. Aeration of the water surrounding the respiration chambers with the respective air/CO_2_ mix was maintained throughout the experimental period to ensure oxygen saturation.

The water inside the respiration chambers circulated permanently at constant velocity by aid of an aquarium pump (8.2 litres min^−1^). A flush pump (5.0 litres min^−1^) facilitated periodic water exchanges between respirometer and its surrounding. *Ṁ*_O_2__ measurement periods of 15 min were alternated with flush periods of 30 min to fully re-establish O_2_ saturation. The O_2_ concentration was determined by optical oxygen probes and recorded using a ten-channel oxygen meter (PreSens-Precision Sensing GmbH, Hamburg, Germany; system 1) as well as a four-channel FireStingO2 (Pyro Science GmbH, Aachen, Germany) and two one-channel Fibox 3 systems (PreSens-Precision Sensing GmbH, Hamburg, Germany) (system 2). For the 0% calibration, the oxygen probes were flushed with nitrogen at room temperature. The calibration for 100% O_2_ was performed in fully aerated water at the respective experimental temperature prior to the measurements of each treatment. Blank measurements to detect bacterial background respiration were recorded following the *Ṁ*_O_2_ _analyses once at every temperature. In order to minimize potential disturbances, all tanks were covered with opaque plastic sheets.

In the respiration chambers, both MMR and SMR were determined at long-term acclimation temperature and *P*_CO_2__. To obtain SMR, individuals remained in the chambers for ∼48 h in order to fully recover from exercise in the swim tunnel. The *Ṁ*_O_2__ values were calculated using the appropriate constants for O_2_ solubility in seawater (Boutilier et al., 1984) and normalized to an average fish weight of 25.6 g following Steffensen et al. (1994). After subtraction of bacterial respiration (solely measurable at 8°C), the first 5 min of the slope of the first *Ṁ*_O_2_ _recording were used to calculate MMR, while the 15% quantile of *Ṁ*_O_2__ recordings starting from the second night in the respiration chamber was considered as SMR (Chabot et al., 2016). After respiration measurements, the length of each fish was measured.

### Calculations and statistical analysis

*Ṁ*_O_2__ data were normalized to an average fish mass (25.6 g) according to Steffensen et al. (1994):
(2)
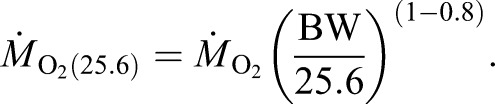
Aerobic scope for exercise (AS_ex_) was defined as:
(3)
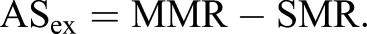
The index of the energetic efficiency of maximum swimming performance (*E*_max_) was calculated as the ratio *U*_crit_ MMR^−1^. Based on concerns outlined by Brett (1962), this index assumes that a potential oxygen debt accumulated during exhaustive burst-type exercise is negligible.

The contribution of anaerobic metabolism (%) during the period between *U*_gait_ and *U*_crit_ was approximated using a duration of one second per burst:
(4)
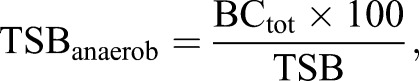
where TSB is time spent bursting (time between *U*_gait_ and *U*_crit_ in s), BC_tot_ is total number of bursts, TSB_anaerob_ is the estimated proportion of anaerobic metabolism between *U*_gait_ and *U*_crit_.

In order to further classify anaerobic swimming performance, we analysed both the maximum consecutive number of bursts at one velocity step and the total number of bursts throughout the whole swim trial.

Individuals that displayed physical abnormalities (*n*=1; 0°C/1170 µatm) or refused to swim (*n*=2; 0°C/390 µatm, 8°C/1170 µatm) because of lethargic behaviour were excluded from data analysis. Fish that refused to swim for no apparent reason (*n*=1; 6°C/1170 µatm) were included in the analysis for SMR. Individuals that did not have any burst capacity were excluded from the statistical analysis for *U*_crit_ for comparability reasons.

Statistical analyses were accomplished using R version 3.0.2 (2013). All variables were tested for normal distribution and homoscedasticity with Shapiro–Wilk and Levene tests, respectively. Owing to heteroscedasticity, the data sets for BC_tot_ and TSB_anaerob_ were log and square root transformed, respectively. Following Nalimov tests, one outlier was removed from the variable AS_ex_ (3°C/390 µatm; *P*=0.0111), *U*_gait_ (3°C/1170 µatm; *P*=0.0007), *E*_max_ (0°C/390 µatm; *P*=0.0073) and TSB_anaerob_ (0°C/390 µatm; *P*=0.0241), respectively. Outlier tests proved inefficient within the one treatment of the variable BC_tot_ (0°C/390 µatm, *P*=0.0233). Therefore, this treatment was tolerated as false positive during further statistical analysis. Statistical comparisons between treatments were performed for the variables SMR, MMR, AS_ex_, *U*_gait_, *U*_crit_, *E*_max_, maximum burst count (BC_max_), BC_tot_, TSB and TSB_anaerob_ using two-way ANOVA. In the case of statistically significant differences, a subsequent *post hoc* Tukey honest significance test was applied. The results of the two-way ANOVA are shown in Table 1, while the results of the Tukey honest significance test between temperature treatments are shown as letters within the figures. Significant differences were assumed using a 5% threshold (*P*<0.05).

**Table 1.**
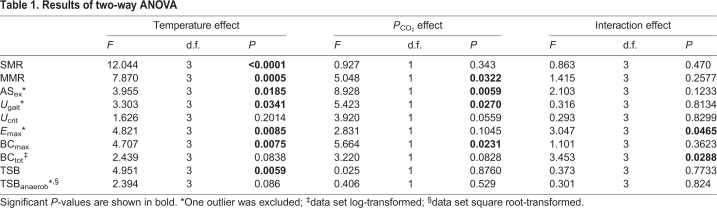
**Results of two-way ANOVA**

The mean number of bursts was expected to increase exponentially with swimming speed. Therefore, we used SigmaPlot 13 (Systat Software Inc., San Jose, California, USA) to find an exponential model with the best fit. The general relationship between swimming speed and burst number at 0, 3 and 8°C was described best by the following model:
(5)
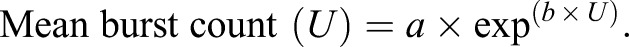


This model was also applied to the data set of 6°C/390 µatm, although the model fit was relatively poor for the data of this treatment (see Table 2 for significance levels). Significant differences in the burst performance between *P*_CO_2__ treatments were accepted in the case of non-overlapping 95% confidence intervals.

**Table 2.**
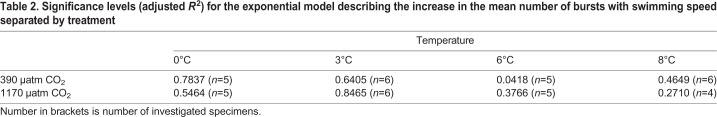
**Significance levels (adjusted *R*^2^) for the exponential model describing the increase in the mean number of bursts with swimming speed separated by treatment**

## RESULTS

Table 3 provides a summary of the results for respiration measurements and swimming performance. A summary of burst swimming parameters is given in Table 4. In total, four individuals showed no bursting event (6°C/390 µatm, *n*=1; 6°C/1170 µatm, *n*=1; 8°C/390 µatm, *n*=2). For a further three individuals (0°C/390 µatm, *n*=1; 6°C/390 µatm, *n*=1; 6°C/1170 µatm, *n*=1), bursting occurred very close to the critical swimming speed (*U*_crit_).

**Table 3.**
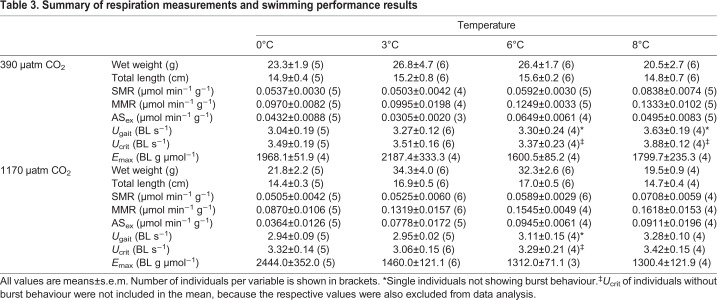
**Summary of respiration measurements and swimming performance results**

**Table 4.**
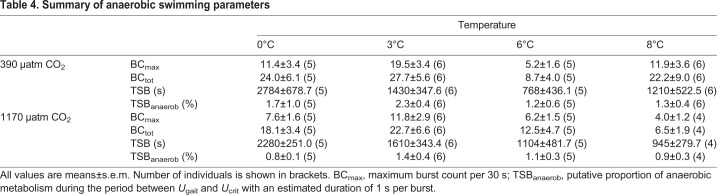
**Summary of anaerobic swimming parameters**

### Respiration

**Fig. 1.**
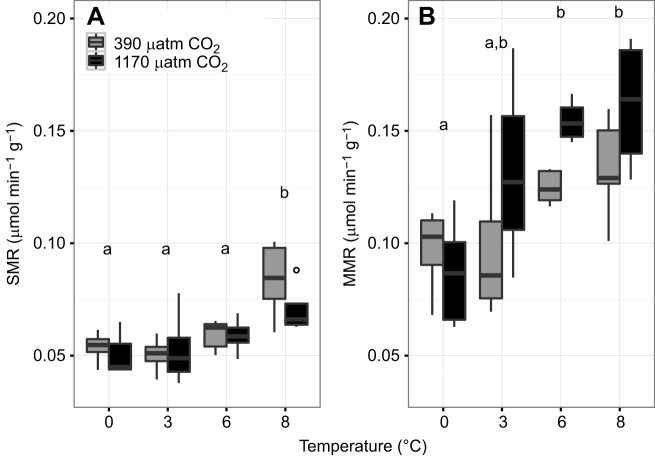
**Standard metabolic rate (SMR) and maximum metabolic rate (MMR) in** **polar cod (*Boreogadus saida*) over a range of acclimation temperatures at** **two *P*_CO_2__ levels.** Boxplots (mean±s.e.m.) of (A) SMR and (B) MMR at four different temperatures and two *P*_CO_2__ levels, as indicated. Full data are summarized in Table 3. Letters indicate results of Tukey honest significance test between temperature treatments (SMR: *P*<0.0001; MMR: *P*=0.0005). Significant differences are represented by different letters. A significant *P*_CO_2__ effect was detected only in MMR (SMR: *P*=0.343; MMR: *P*=0.0322); no interaction effect (two-way ANOVA) was observed (SMR: *P*=0.470; MMR: *P*=0.2577) (see Table 1). All boxes show 25th and 75th percentiles with median; whiskers are 5th and 95th percentiles.

**Fig. 2.**
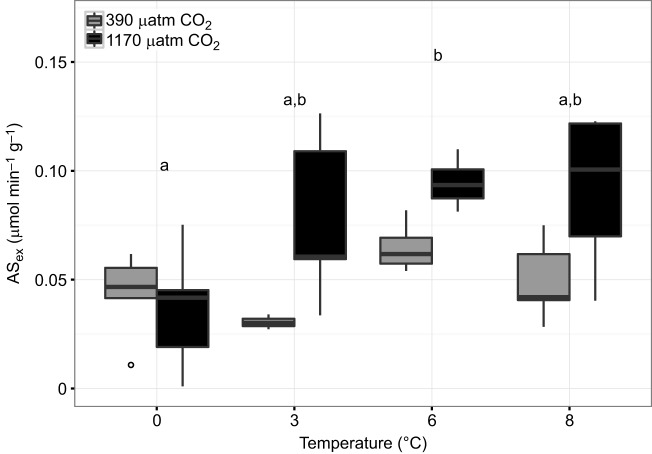
**Aerobic scope (AS_ex_) for exercise in** ***B.*** ***saida* over a range of temperatures at two *P*_CO_2__ levels.** Boxplots (mean±s.e.m) of AS_ex_ at four different temperatures and two *P*_CO_2__ levels, as indicated. Full data are summarized in Table 3. Letters indicate results of Tukey honest significance test between temperature treatments (*P*=0.0185). Significant differences are represented by different letters. A significant *P*_CO_2__ effect was detected (*P*=0.0059); no interaction effect (two-way ANOVA) was observed (*P*=0.1233) (see Table 1).

The SMR of *B. saida* showed comparable values at 0, 3 and 6°C, but was significantly higher at 8°C (0°C versus 8°C, *P*<0.0001; 3°C versus 8°C, *P*<0.0001; 6°C versus 8°C, *P*=0.0005). Hypercapnia did not reveal any effect on the SMR of this species (*P*=0.342) (Fig. 1A). Long-term acclimation to different temperatures had a distinct effect (*P*=0.0005) on the MMR of *B. saida*: MMR rose significantly between 0 and 6°C (*P*=0.0041), where it levelled off (6°C versus 8°C, *P*=0.9232). At all temperatures but 0°C, MMR was enhanced in high *P*_CO_2__ treatments compared with control *P*_CO_2_ _treatments (*P*=0.0322) (Fig. 1B). An overall temperature effect (*P*=0.0185) was recorded for the aerobic scope of exercise (AS_ex_) after 4 months with a peak observed at 6°C (0°C versus 6°C, *P*=0.0336). Furthermore, AS_ex_ was significantly elevated under high *P*_CO_2__ conditions (*P*=0.0059) (Fig. 2).

### Swimming performance

**Fig. 3.**
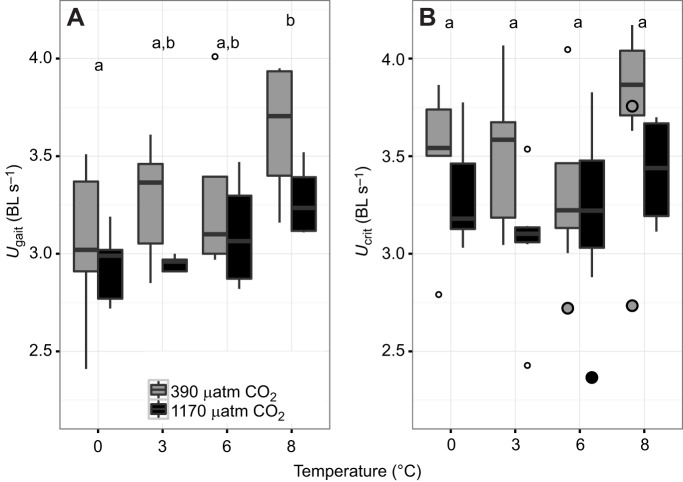
**Gait transition speed (*U*_gait_) and critical swimming speed (*U*_crit_) in *B. saida* over a range of acclimation temperatures at two *P*_CO_2__ levels. ** Boxplots (mean±s.e.m.) of (A) *U*_gait_ and (B) *U*_crit_ (adjusted according to Brett, 1964) at four different temperatures and two *P*_CO_2__ levels, as indicated. Filled circles are *U*_crit_ of individuals without burst capacity (not included in statistical analysis). Full data are summarized in Table 3. Letters indicate results of Tukey honest significance test between temperature treatments (*U*_gait_: *P*=0.0341; *U*_crit_: *P*=0.2014). Significant differences are represented by different letters. A significant *P*_CO_2__ effect was solely detected in *U*_gait_ (*U*_gait_: *P*=0.0270; *U*_crit_: *P*=0.0559); no interaction effect (two-way ANOVA) was observed (*U*_gait_: *P*=0.8134; *U*_crit_: *P*=0.8299) (see Table 1).

The transition speed from purely aerobic to partly anaerobic swimming performance (*U*_gait_) increased significantly with long-term acclimation temperature (*P*=0.0341). The significant difference, nevertheless, refers to an elevated *U*_gait_ at the highest (8°C) compared with the lowest acclimation temperature (0°C), indicating an overall modest temperature effect on this parameter. Long-term acclimation at high *P*_CO_2__ significantly depressed *U*_gait_ (*P*=0.0270; Fig. 3A). An interaction between temperature and *P*_CO_2__ level was not found for this parameter (*P*=0.8134). *U*_crit_ did not reveal a significant temperature effect (*P*=0.2014). *U*_crit_ data, however, indicated a downward trend due to hypercapnia (*P*=0.0559; Fig. 3B).

**Fig. 4.**
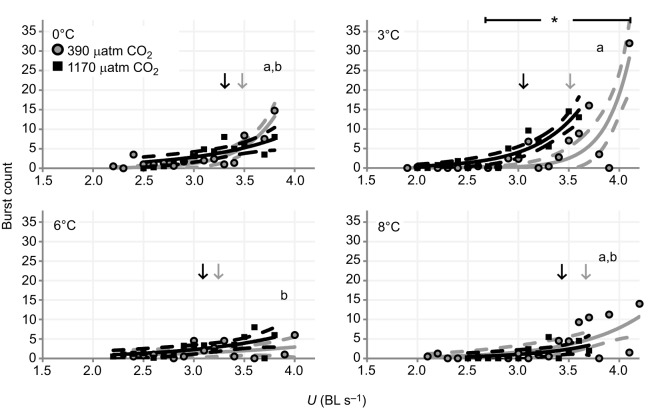
**Mean number of bursts per treatment per velocity step (BL s^−1^) in** ***B.*** ***saida* over a range of acclimation temperatures at two *P*_CO_2__ levels.** Arrows indicate mean *U*_crit_ per treatment (note that *U*_crit_ values of individuals without burst capacity were included in the calculation of mean *U*_crit_). Solid lines represent a data fit to a nonlinear regression [mean burst count (*U*) = *a×exp*(*U×b*)]. Dashed lines are 95% confidence intervals (CI) of nonlinear regression. Asterisk indicates significant *P*_CO_2__ effect (non-overlapping 95% CI). Letters show results of Tukey honest significance test for maximum burst count (BC_max_) between temperature treatments (*P*=0.0075). Significant differences are represented by different letters. A significant *P*_CO_2__ effect was detected for BC_max_ (*P*=0.0231); no interaction effect (two-way ANOVA) was observed for BC_max_ (*P*=0.3623) (see Table 1).

The maximum number of bursts, a parameter assumed to reflect the capacity for anaerobic swimming, was highest at 3°C (0°C versus 3°C, *P*=0.1358; 3°C versus 6°C, *P*=0.0051; 3°C versus 8°C, *P*=0.0776). At the same time, the maximum burst count was found to be higher under normocapnia than hypercapnia (*P*=0.0231). Furthermore, elevated *P*_CO_2__ shifted mean burst performance at 3°C to lower velocities under hypercapnia (non-overlapping 95% CI; asterisk in Fig. 4). In contrast to the results for maximum number of bursts, neither temperature nor *P*_CO_2__^ ^affected the total number of bursts significantly. However, a combined effect of temperature and *P*_CO_2__ level was detected (*P*=0.0288), mainly evoked by the low number of bursts detected in the treatment at 8°C/1170 µatm. The time between *U*_gait_ and *U*_crit_ – hereafter classified as ‘time spent bursting’ (TSB) – revealed a decreasing trend with temperature (*P*=0.0059), with no apparent *P*_CO_2__ effect (*P*=0.8760). The putative contribution of anaerobic metabolism to swimming performance between *U*_gait_ and *U*_crit_ (TSB_anaerob_) was low overall (<3% in 92.9% of individuals) with no apparent influence of temperature or *P*_CO_2__ (Table 4).

**Fig. 5.**
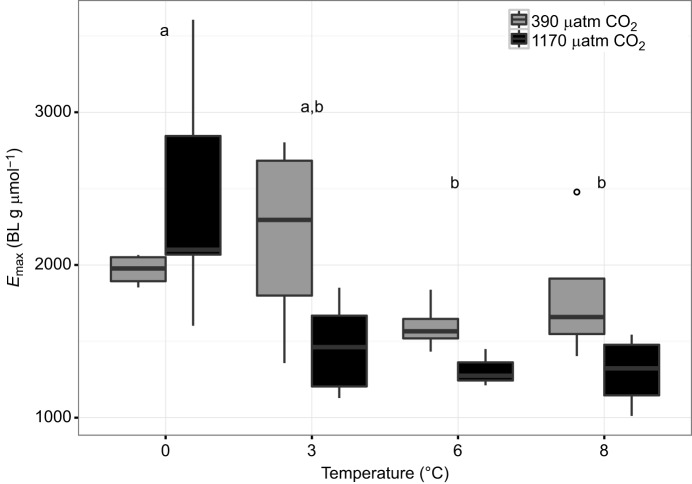
**Efficiency of maximum swimming performance (*E*_max_) in** ***B.saida* over a range of acclimation temperatures at two *P*_CO_2__ levels.** Boxplots (means±s.e.m.) of *E*_max_ at four different temperatures and two *P*_CO_2__ levels, as indicated. Full data are summarized in Table 3. Letters indicate results of Tukey honest significance test between temperature treatments (*P*=0.0085). Significant differences are represented by different letters. No significant difference between *P*_CO_2__ treatments was found (*P*=0.1045). An interaction effect (two-way ANOVA) was observed (*P*=0.0465) (see Table 1).

Energetic efficiency of maximum swimming performance (*E*_max_) (Fig. 5) was high at 0°C (0°C versus 3°C, *P*=0.1161; 0°C versus 6°C, *P*=0.0128; 0°C versus 8°C, *P*=0.0210). Although no significant impact of hypercapnia was detected (*P*=0.1045), *E*_max_ was reduced under high *P*_CO_2__ conditions at all temperatures above 0°C due to the elevated MMR under hypercapnic conditions between 3 and 8°C. Furthermore, *E*_max_ showed a significant interaction effect of temperature and *P*_CO_2__ evoked by a strongly elevated value at 0°C under hypercapnia (0°C/1170 µatm versus 3°C/1170 µatm, *P*=0.0236; 0°C/1170 µatm versus 6°C/1170 µatm, *P*=0.0348; 0°C/1170 µatm versus 8°C/1170 µatm, *P*=0.0155).

## DISCUSSION

The present study aimed to investigate oxygen consumption and exercise capacities of *B. saida* after long-term acclimation to future OAW conditions in order to estimate the competitive strength of this species under future environmental conditions at *P*_CO_2__ levels following the RCP8.5 scenario (IPCC, 2014). Our results suggest that enhanced costs visible in elevated MMR under hypercapnic water conditions cause a reduction in maximum swimming capacity.

At comparable temperatures, the SMR obtained in the present study was in the same order of magnitude as published for *B. saida* (Steffensen et al., 1994, 4.5°C; Drost et al., 2016, 1.0, 3.5, 6.5°C), when applying the *Ṁ*_O_2__ units and the weight correction formula of the respective studies. Slight deviations in SMR are likely to be attributable to divergent approaches to determine SMR: when applying the same approach for the determination of SMR as used by Drost et al. (2016) (assuming the lowest *Ṁ*_O_2__ recording as SMR) for comparison between both studies, the resulting SMR values of the present study (65, 64 and 78 mg O_2_ kg^−1^ h^−1^ at 0, 3 and 6°C, respectively) are fairly similar to those published by Drost et al. (2016) (∼53, 50 and 76 mg O_2_ kg^−1^ h^−1^ at 1, 3.5 and 6.5°C, respectively). The approach chosen by Drost et al. (2016) was the only method applicable to their particular experimental design. However, Chabot et al. (2016) raised the concern of an underestimation of SMR due to temporal variability within this parameter when only a single *Ṁ*_O_2__ measurement is chosen to represent SMR. In order to correct for temporal variability, we preferred to calculate SMR by aid of a quantile approach allowing 15% of the resting *Ṁ*_O_2__ values to fall below the actual individual SMR (Dupont-Prinet et al., 2013; Chabot et al., 2016).

The SMRs of *B. saida* acclimated long-term at 3 and 6°C were similar to those found in the 0°C acclimated individuals. This implies efficient metabolic compensation in the thermal range between 0 and 6°C (Precht, 1958). Metabolic compensation is hypothesized to enable the individual to maintain vital functions independent of environmental temperatures (Precht, 1958). Hop and Graham (1995) detected incomplete compensation in the SMR of *B. saida* following a 12 day exposure to 2.7°C compared with SMR values obtained in specimens acclimated for 5 months at 0.4°C. Accordingly, the rather short exposure period (12 days) to the elevated temperature was probably insufficient to establish a new physiological steady state and thereby to unfold the full acclimation potential of this species. At 8°C, the SMR of *B. saida* was significantly elevated, even after 4 months of acclimation, also perceived in a non-significantly reduced growth performance (Kunz et al., 2016a). Mitochondrial plasticity has been identified to be involved in setting the limits of thermal acclimation capacity (e.g. Strobel et al., 2013). Accordingly, the elevated whole-animal SMR at the highest temperature investigated may, at least partly, be attributed to limited acclimation capacities expressed through reduced mitochondrial efficiencies at 8°C shown in cardiac myocytes of the same individuals as used in the present study (Leo et al., 2017). *B. saida* revealed little capacity to adjust mitochondrial enzyme activities and lipid class compositions in response to warm acclimation above 6°C (Elettra Leo, Martin Graeve, Daniela Storch, H.-O.P. and F.C.M., unpublished data). Accordingly, an enhanced proton leak and an associated decrease in ATP production efficiency evoked by changes in membrane fluidity are suggested to cause the impaired mitochondrial efficiency in *B. saida* close to its long-term whole-animal upper thermal tolerance limit [pejus temperatures (*T*_pej_) *sensu* Pörtner et al., 2017; Leo et al., 2017].

Elevated CO_2_ levels did not influence the SMR of *B. saida* in the present study in line with findings for the Atlantic cod (*Gadus morhua*) (3–4 weeks of exposure, Kreiss et al., 2015) and the Antarctic *Notothenia rossii* (29–36 days of exposure, Strobel et al., 2012), long-term acclimated to moderate *P*_CO_2__ conditions. This suggests a rather low sensitivity of baseline metabolism to hypercapnia. Accordingly, the resting cardiac mitochondrial respiration of *B. saida* was not affected by chronically elevated *P*_CO_2__ (Leo et al., 2017).

Recorded values for MMR are consistent with recently published results for *B. saida* (Drost et al., 2016). A positive correlation between MMR and environmental temperatures as detected in the present study is well established for diverse teleost species (e.g. Claireaux et al., 2006; Eliason et al., 2011; Clark et al., 2011) along with an increase in cardiorespiratory performance with temperature. Limitations in heart rate and oxygen-carrying capacity are hypothesized to cause a levelling off in MMR at high acclimation temperatures (Pörtner, 2010), as seen in the present study.

The continuous increase in MMR with temperature and the elevated SMR at 8°C result in a peak aerobic scope for exercise (AS_ex_) of *B. saida* acclimated to 6°C. In line with this observation, growth of *B. saida* under laboratory conditions was also maximum at 6°C (Kunz et al., 2016a), suggesting a connection between aerobic capacities and growth as well as exercise (Pörtner and Knust, 2007; Pörtner and Farrell, 2008). The optimization of aerobic performance governed by environmental factors is widely recognized to determine a species' spatial and temporal niche (Claireaux and Lagardère, 1999; Pörtner and Farrell, 2008). Nevertheless, fish often inhabit areas with temperatures well below their physiological optimum obtained under artificial *ad libitum* food situations (Björnsson et al., 2001) indicating that maximum exploitation of aerobic scope is not a precondition for survival (Deutsch et al., 2015; Norin and Clark, 2016). Despite cold-induced reductions in AS_ex_ due to lower MMR, *B. saida* is well adapted to temperatures around 0°C, visible in a high feed conversion efficiency (Kunz et al., 2016a). Likewise, Hop et al. (1997) found assimilation efficiencies at 0°C (average 80%), similar to assimilation capacities detected in stenothermal Antarctic fish. Thus, *B. saida* likely has an energetic advantage over potential predators and competitors in cold waters. In contrast, when inhabiting waters with their maximum AS_ex_ and their optimum temperature for growth (6°C) (Kunz et al., 2016a), a reduced abundance of suitable prey (Fossheim et al., 2015) would probably demand elevated energy fractions for foraging activity, possibly constraining growth and reproduction. Accordingly, during ongoing climate change, polar fish at their southern distribution limits such as *B. saida* may be especially vulnerable to competition with invading species adapted to higher water temperatures. Among the northwards moving species, capelin (*Mallotus villosus*), Atlantic cod and haddock (*Melanogrammus aeglefinus*) represent a potential treat to *B. saida*. While *M. villosus* is expected to compete for prey with *B. saida* during a progressive future habitat overlap (Hop and Gjøsæter, 2013), juvenile *G. morhua* and *M. aeglefinus* revealed little dietary overlap with *B. saida* in habitats where they co-occurred (Renaud et al., 2012). During ongoing climate change, however, adult *G. morhua* and *M. aeglefinus* may become increasingly important as predators on *B. saida* (Renaud et al., 2012). Nevertheless, the distribution of *B. saida* has already been observed to contract in its southern habitat as a direct result of increasing water temperatures (Eriksen et al., 2015).

In parallel to AS_ex_, both mainly aerobically fuelled steady-state swimming performance (Lurman et al., 2007) (*U*_gait_) and partly anaerobically fuelled *U*_crit_ are known to increase acutely with temperature up to maximum performance before decreasing at temperatures approaching the critical thermal limit (*T*_C,max_ *sensu* Farrell, 2016) (e.g. Griffiths and Alderdice, 1972). In the present study, however, *U*_gait_ of *B. saida* showed only a modest increase with acclimation temperature. *U*_crit_ did not reveal any clear trend with long-term acclimation temperature. These results indicate that metabolic compensation processes during warm acclimation as observed for the SMR of *B. saida* (see above) were also reflected in swimming metabolism for this species. Thus, while *U*_gait_ and *U*_crit_ of *B. saida* showed signs of acclimation throughout the range of investigated temperatures (0–8°C), the SMR of *B. saida* showed full compensation up to only 6°C attributed to a significant reduction in mitochondrial ATP production efficiency at 8°C compared with lower acclimation temperatures (0, 3 and 6°C) (Leo et al., 2017). The decreasing mitochondrial ATP production efficiency may further contribute to the observed decrease in muscle output efficiency (here expressed as *E*_max_) with acclimation temperature. Based on the reduced mitochondrial efficiencies that translated into organismic limitations, we expect an overall limited capacity of *B. saida* to acclimate to water conditions higher than 6°C. Similar to indications obtained in our study, Drost et al. (2016), who investigated the thermal acclimation response of *B. saida* from the Canadian Arctic by measuring cardio-respiratory performance, found that *B. saida* can acclimate to 6.5°C (highest investigated acclimation temperature). Nevertheless, cardio-respiratory limitations caused a higher sensitivity of 6.5°C-acclimated specimens to acute temperature changes compared with *B. saida* acclimated to lower temperatures (Drost et al., 2016).

The switch from aerobic to anaerobic metabolism at *U*_gait_ is marked by burst-type exercise events (Milligan and Wood, 1986; Lurman et al., 2007). Burst performance is essential during predator–prey interactions (Beamish, 1978), and can only be maintained for short periods. *B. saida* showed low burst capacity throughout all temperature/*P*_CO_2__ treatments (Table 4), with a few specimens (*n*=4 at 6 and 8°C treatments out of in total 42 specimens used in the swimming performance tests) not even displaying any burst behaviour at all. Accordingly, the contribution of anaerobic metabolism to maximum swimming capacity is putatively minor. This phenomenon is in line with observations in other polar species, including Antarctic fishes that revealed low potential for anaerobic glycolysis (e.g. the yellowbelly rockcod *Notothenia neglecta*, Dunn and Johnston, 1986; the bald notothen *Pagothenia borchgrevinki*, Davison et al., 1988). Compared with active temperate species, the burst performance of *B. saida* is several-fold lower (highest maximum burst count for *B. saida*: 19.5 for 30 s, 3°C versus e.g. European sea bass *Dicentrarchus labrax*, 23°C; maximum burst count at *U*_crit_: ∼84 for 30 s, Marras et al., 2010). Furthermore, the stores of the white muscle metabolites ATP and glycogen have been shown to remain independent of acclimation temperature (see review by Kieffer, 2000). Combined with the low level of anaerobically fuelled swimming capacity (0–5.7%), the slightly elevated burst performance (both BC_max_ and BC_tot_) of *B. saida* at 3°C found in the present study is not given much weight. Hence, the moderately active lifestyle of *B. saida* described by Gradinger and Bluhm (2004) is also mirrored in the low-burst swimming performance, possibly involving a disadvantage during predator avoidance.

The response of aerobic capacities to near-future elevated *P*_CO_2__ conditions has been found to be strongly species specific (compare review by Esbaugh, 2018), with reduced sensitivities to CO_2_ suggested for species frequently exposed to natural fluctuations in abiotic conditions (Rummer et al., 2013). The majority of studies did not detect any impact of near-future *P*_CO_2__ conditions on MMR in a variety of species after chronic exposure (*G. morhua*, 4 months at 3000 µatm, Melzner et al., 2009; red drum, *Sciaenops ocellatus*, 14 days at 1000 µatm, Esbaugh et al., 2016; blue rockfish, *Sebastes mystinus* 16–19 weeks at 750, 1900 and 2800 µatm, Hamilton et al., 2017). In contrast, a reduction of MMR under ocean acidification was found in copper rockfish (*Sebastes caurinus*) exposed to 1900 µatm CO_2_ and 10°C for 14–17 weeks (Hamilton et al., 2017). *B. saida*, however, revealed an elevated MMR under near-future *P*_CO_2__ conditions (at all temperatures above 0°C), in line with observations in the tropical coral reef fish *Acanthochromis polyacanthus* (17 days at 950 µatm, Rummer et al., 2013) and the temperate species *D. labrax* under long-term exposure to realistic *P*_CO_2__ scenarios (1.5 years, 1500 µatm, Amélie Crespel, Katja Anttila, Pernelle Lelièvre, Patrick Quazuguel, Nicolas Le Bayon, José-Luis Zambonino-Infante, Denis Chabot and G.C., unpublished data). Interestingly, *B. saida* showed reduced maximum swimming velocities despite elevated MMR. Unfortunately, the studies of Rummer et al. (2013) and Crespel and coworkers (unpublished data) do not report on swimming performance. As a consequence of elevated MMR and reduced swimming performance, *E*_max_ was impaired in *B. saida* under hypercapnia at all temperatures above 0°C. Under control *P*_CO_2__ conditions, however, swimming performance efficiency was only reduced at acclimation temperatures above 3°C, suggesting a higher thermal sensitivity of MMR under hypercapnia. Nevertheless, *E*_max_ has to be considered with care as an unquantifiable oxygen debt may interfere with it (Brett, 1962), which, in turn, is expected to be of little extent due to limited anaerobic capacities implied by a low burst capacity detected in the present study.

To date, physiological mechanisms causing elevated aerobic metabolic costs under moderate *P*_CO_2__ conditions are not fully understood. Our results suggest that this cost is likely elevated through mechanisms other than exercise and constrains swimming performance (both *U*_gait_ and *U*_crit_) to lower levels under hypercapnia while causing elevated *Ṁ*_O_2__ and a consequently higher AS_ex_. One organ potentially being involved in elevated energy demands under high *P*_CO_2__ conditions is the gill. Kreiss et al. (2015) found branchial *Ṁ*_O_2__ per gram gill tissue after long-term acclimation of *G. morhua* to 2200 µatm remained comparable to values under control *P*_CO_2__ conditions at 10°C. However, high *P*_CO_2__ caused an increase in gill soft tissue, resulting in elevated fractions of gill *Ṁ*_O_2__ from whole-animal *Ṁ*_O_2__ (increase from 5 to 7%) (Kreiss et al., 2015). Nevertheless, the SMR of both *G. morhua* (Kreiss et al., 2015) and *B. saida* (present study) remained unaffected under hypercapnia. Yet, potential cascading effects might be amplified during maximum performance causing trade-offs in swimming capacity as seen in *B. saida*. The results of the present study focusing on whole-animal parameters, however, represent only an ensemble of organismic costs and do not give further insight into the energetic partition of underlying mechanisms.

Hypercapnia-acclimated fish not only showed a shift to anaerobic white muscle reserves at lower swimming speeds (as observed by lower *U*_gait_), but the burst performance was also reduced compared with normocapnia-acclimated fish. Despite a marginal contribution of anaerobic metabolism to the maximum swimming capacities of *B. saida*, this finding is in line with the overall impairment in maximum performance detected following exposure to elevated *P*_CO_2__ levels. Hence, aerobic as well as anaerobic exercise capacities appear reduced under high *P*_CO_2__ scenarios. Thus, hypercapnia has effects on the energy metabolism of *B. saida* at high and maximum metabolic rates that are not visible at rest (Kunz et al., 2016a).

In conclusion, the present study revealed a strong impact of ocean acidification on maximum performance traits of *B. saida*. Although elevated *P*_CO_2__ levels did not significantly impact routine parameters (growth, food consumption, SMR) in this species (Kunz et al., 2016a), trade-offs in energy allocation became visible when the metabolism was operating at maximum performance under hypercapnic conditions. More precisely, long-term acclimation under near-future *P*_CO_2__ conditions caused reduced swimming capacity of *B. saida* at higher metabolic costs. Consequently, when translating the present results obtained from a limited number of specimens onto the population level, foraging success and escape response of *B. saida* during predator encounters might be impaired under future water conditions. Species that are resilient to a broader range of abiotic conditions, such as *G. morhua* (Melzner et al., 2009), may find it easier to prevail in light of the ongoing borealization and community shifts in the Arctic (Fossheim et al., 2015). Accordingly, the competitive strength of *B. saida*, and thereby its abundance in this new setting in the waters around Svalbard can be expected to decrease.
